# Transformer fault diagnosis using continuous sparse autoencoder

**DOI:** 10.1186/s40064-016-2107-7

**Published:** 2016-04-14

**Authors:** Lukun Wang, Xiaoying Zhao, Jiangnan Pei, Gongyou Tang

**Affiliations:** College of Information Science and Engineering, Ocean University of China, Qingdao, China; College of Foreign Languages, Taishan Medical University, Taian, China; College of Electrical Engineering and Automation, Shandong University of Science and Technology, Qingdao, China

**Keywords:** Continuous sparse autoencoder, Dissolved gas analysis, Deep belief network, Deep learning, Transformer fault

## Abstract

This paper proposes a novel continuous sparse autoencoder (CSAE) which can be used in unsupervised feature learning. The CSAE adds Gaussian stochastic unit into activation function to extract features of nonlinear data. In this paper, CSAE is applied to solve the problem of transformer fault recognition. Firstly, based on dissolved gas analysis method, IEC three ratios are calculated by the concentrations of dissolved gases. Then IEC three ratios data is normalized to reduce data singularity and improve training speed. Secondly, deep belief network is established by two layers of CSAE and one layer of back propagation (BP) network. Thirdly, CSAE is adopted to unsupervised training and getting features. Then BP network is used for supervised training and getting transformer fault. Finally, the experimental data from IEC TC 10 dataset aims to illustrate the effectiveness of the presented approach. Comparative experiments clearly show that CSAE can extract features from the original data, and achieve a superior correct differentiation rate on transformer fault diagnosis.

## Background

Transformer is one of the most important equipment in power network. It will bring huge economic loss to the power network if it fails. The periodical monitoring of the condition of the transformer is necessary. There are a lot of methods used for detecting power failures such as oil breakdown voltage test, resistivity test and moisture analysis in transformer oil (Saha 2003). Among these methods, dissolved gas analysis (DGA) is the most widely used method (Arakelian 2004). This method diagnoses the transformer fault based on the analysis of dissolved gas concentrations in transformer oil (Duval 2003). The gases in transformer oil mainly include hydrocarbons, such as: methane (CH_4_), ethane (C_2_H_6_), ethylene (C_2_H_4_), acetylene (C_2_H_2_) and other gases, such as: hydrogen (H_2_) and carbon dioxide (CO_2_). In recent years, researchers have proposed transformer fault diagnosis methods including particle swarm optimization (Ballal et al. 2013), support vector machine (Chen et al. 2009), fuzzy learning vector quantization network (Yang et al. 2001) and back propagation (BP) neural network (Patel and Khubchandani 2004). Miranda et al. (2012) built a diagnosis system based on a set of auto-associative neural networks to diagnose the faults of power transformer. The information theoretic mean shift (ITMS) algorithm was adopted to densify the data clusters. Dhote and Helonde (2012) proposed a new five fuzzy ratios method and developed a fuzzy diagnostic expert system to diagnose the transformer fault. Souahlia et al. (2012) combined the Rogers and Doernenburg ratios together to be the gases signature. The multi-layer perceptron neural network was applied for decision making. Bhalla et al. (2012) applied a pedagogical approach for rule extraction from function approximating ANN (REFANN). REFANN derives linear equations by approximating the hidden unit activation function and splitting the input space into sub-region. Ren et al. (2010) used the rough set theory to reduce the degree of complex training samples; the speed of learning and training was enhanced. Then the quantum neural network was applied to the classifier of transformer fault diagnosis.

In 1996, sparse coding was proposed by Olshausen and Field (1996) which showed that the receptive fields of simple cells in mammalian primary visual cortex could learn higher level representations from the outside input signals (Vinje and Gallant 2000). After then, autoencoder was proposed to learn higher level features. In 2006, a new neural network model called deep belief network (DBN) was proposed by Hinton and Salakhutdinov (2006) as a new neural network (Cottrell 2006). With the development of the deep learning theory, DBN is widely used in many AI areas (Le Roux and Bengio 2010).

According to Bengio et al. (2006), DBN was successfully comprised of autoencoder (AE). He used AE as a basic model of DBN. With this structure, the training of handwritten digits recognition has achieved more than 99 % accuracy rate. It is proved that AE can completely replace restricted Boltzmann machine (RBM) as the basic elements of DBN. In 2008, Vincent et al. (2008) proposed denoising autoencoder (DAE) which could be adopted in corrupted data. DAE learns to project the corrupted data back onto the manifold, and can make the characteristics of the data more robust. On this basis, Vincent et al. (2010) introduced stacked denoising autoencoder (SDAE) by stacking several layers of DAE with the category constraint. At present, AE has been successfully applied to speech recognition (Dahl et al. 2012), handwritten digit recognition, natural language processing fields (Glorot et al. 2011), etc.

The current research on transformer fault diagnosis which applies neural network to the classification algorithm is mainly based on single-layer neural network. Instead of a single-layer neural network, a deep network composed of multiple layers of continuous sparse autoencoder (CSAE) is designed to solve the problem of transformer fault recognition. The second section describes the method of DGA, the relationship between the transformer fault classification and the concentrations of five fault gases has been introduced. In the third section, the basic autoencoder is briefly reviewed and a new continuous sparse autoencoder is proposed to extract the features of nonlinear data. The fourth section, several experiments are designed to verify the validity of CSAE. The last section concludes our work and points out the future direction.

## Dissolved gas analysis

DGA is an analytic technique by detecting the dissolved gas in transformer oil. The insulating materials will release small amounts of hydrocarbons if transformer breaks down. The concentrations of these hydrocarbons can be used for electrical fault classification. The gases generated by transformer have useful information. They can be applied to electrical equipment diagnosis.

IEC publication 60599 (Duval 2003) provided a list of faults for DGA. The common transformer faults and their symbols are shown in Table 1.

**Table 1:** Fault classification

Symbol	Transformer fault
PD	Partial discharges
LED	Low energy discharge
HED	High energy discharge
TF1	Thermal faults <700 °C
TF2	Thermal faults >700 °C

Under the influence of thermal faults and electrical faults, hydrocarbon molecules of mineral oil can be decomposed from active hydrogen and hydrocarbon fragments. Then small amounts of gases, such like H_2_, CH_4_, C_2_H_6_, C_2_H_4_, and C_2_H_2_ will be released. The emergence of these gases often accompanies with transformer faults, therefore these five gases are named as fault gases. The fault gases are released in the following order: H_2_ → CH_4_ → C_2_H_6_ → C_2_H_4_ → C_2_H_2_. The concentration of hydrogen will increase steadily when the temperature is relatively low, while the acetylene will be released at a very high temperature. Therefore, the fault gases keep in touch with transformer fault. The relationship between the concentration of fault gases and transformer fault is shown in Table 2. In electrical faults, hydrogen is of high importance, while in thermal faults, acetylene tends to be important. In low thermal faults, methane, ethane and ethylene is of high importance but in high thermal faults only ethylene tends to be of high importance. The main difference between low thermal faults and high thermal faults is the concentration of thane. Ethane will be released when low thermal faults happen. According to the analysis above, DGA can diagnose the transformer fault by detecting the concentrations of these five fault gases.

**Table 2:** Gas importance by faults

Cause of gas generation	H_2_	CH_4_	C_2_H_6_	C_2_H_4_	C_2_H_2_
Electrical fault					
PD	●	○			
LED	●				●
HED	●			○	●
Thermal fault					
TF1	○	●	●	●	
TF2	○	○		●	○

●: high importance, ○: medium importance

## Methods

### DBN model

**Fig. 1:**
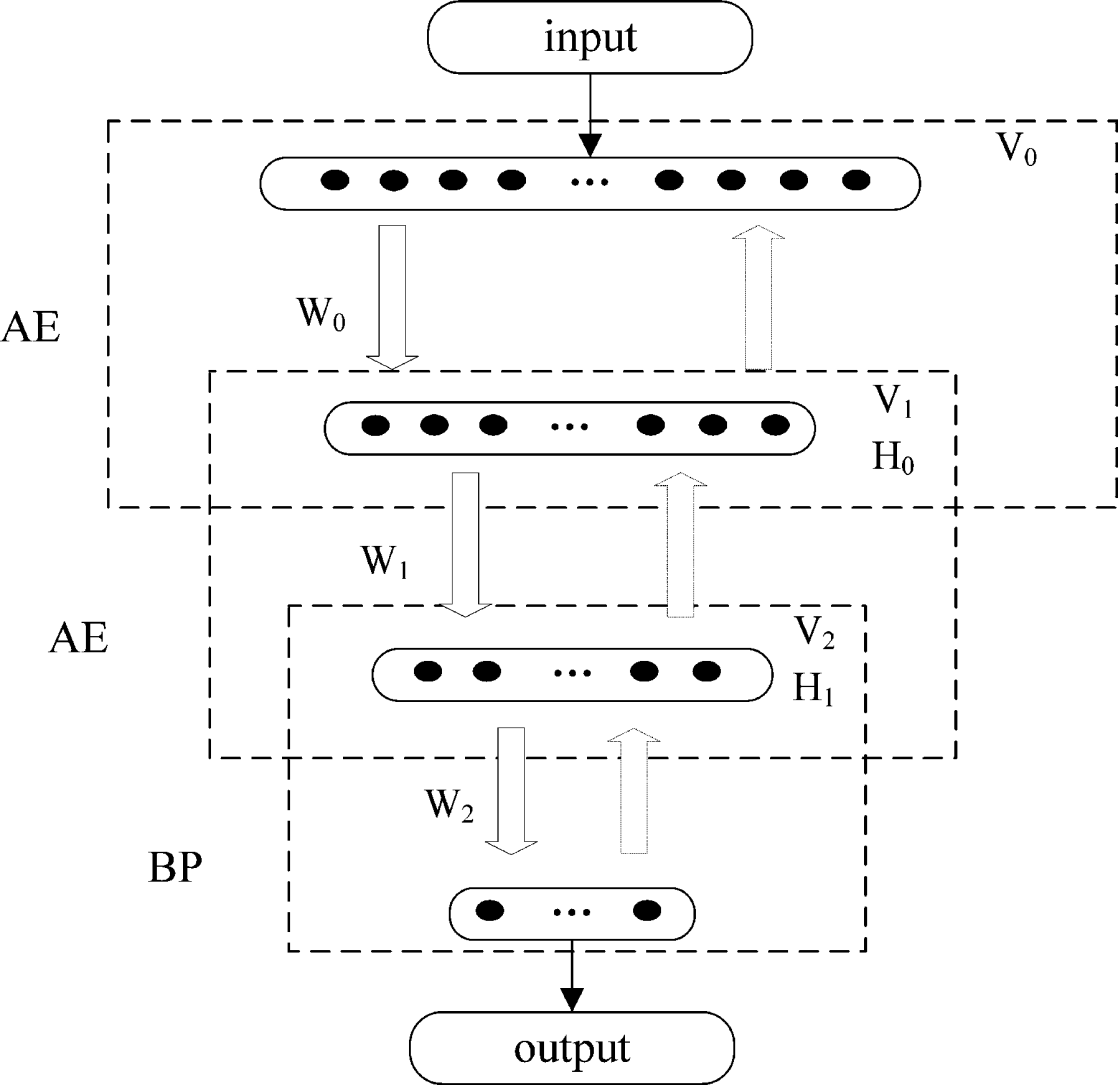
DBN model

DBN is a logic model consisted of multiple layers of RBM. It also can be composed of multiple layers of AE. The structure of DBN based on multiple layers of AE is shown in Fig. 1.

The process of training DBN can be divided into the following steps:

- *Step 1* Each layer of AE can be used for unsupervised feature learning. In the process of training, each layer of AE can extract different features from the input data. These features are stored in the feature vector *W*. In this step, the optimization is not meant for the entire DBN.
- *Step 2* One layer of BP neural network is set at the bottom layer of DBN. The reason of setting one layer of BP is to receive trained AE weight. After AE unsupervised training, BP will calculate the error between DBN output and expected output. The error will be passed back to previous layers of AE. According to the error, the weight matrix of the whole DBN will be updated. The process of reconstruction will be repeated based on the set epochs until the error converges. It realizes the optimization of feature data.

DBN overcomes the disadvantages of signal-layer neural network: falling into local optimum and long training time.

### Basic autoencoder

**Fig. 2:**
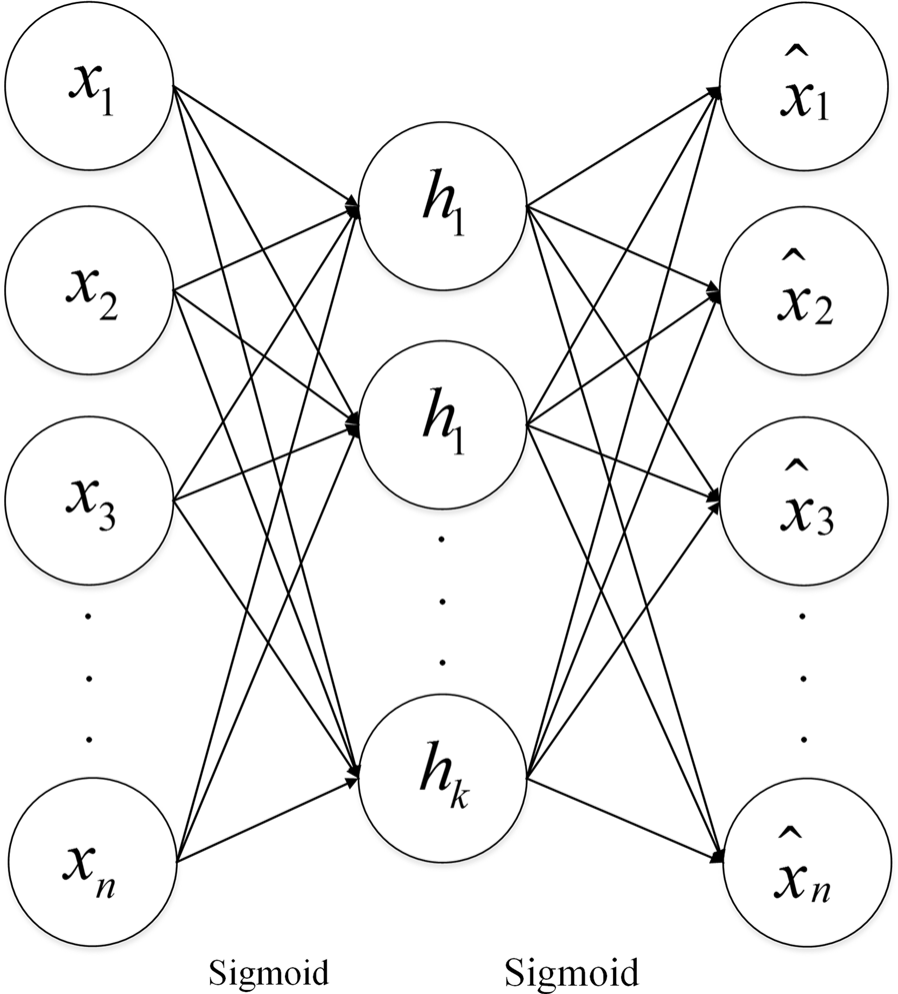
Model of AE

Autoencoder is a famous neural network model in which the target output is as same as the input, such as $y^{\left( i \right)} = x^{\left( i \right)}$. Autoencoder has two processes: encoder process and decoder process. In the encoder process, the input is transformed into the hidden features. In the decoder process, the hidden features are reconstructed to be the target output. The weight matrix of each layer can be updated through training neural network. The structure is shown in Fig. 2.

Where $x_{i},i \in 1,\ldots,n$ is the input of autoencoder, $h_{j},j \in 1,\ldots,k$ is the value of hidden units, $\hat{x}_{i},i \in 1,\ldots,n$ is the target output, $W^{(i)} ,i \in 1,2$ denotes the weight matrix. AE tries to learn a function like $h_{W,b} \left( x \right) = x$ which can make $\hat{x}$ approximate to *x*. $h_{W,b} \left( x \right)$ is an activation function. The purpose of training AE is to get $\left\{ {W^{\left( l \right)} ,b^{\left( l \right)} } \right\}$.

In order to acquire the weight matrix, the square error of single sample can be calculated as

1 $$J\left( {W,b;x,y} \right) = \frac{1}{2}\left\| {h_{W,b} \left( x \right) - y} \right\|^{2}$$

where $x$ and $y$ denote the real input and output respectively, $h_{W,b} \left( x \right)$ is the output of activation function.

The error loss function of whole network can be obtained

2 $$\begin{aligned} J\left( {W,b} \right) &= \left[ {\frac{1}{m}\mathop \sum \limits_{i = 1}^{m} J\left( {W,b;x^{\left( i \right)} ,y^{\left( i \right)} } \right)} \right] + \frac{\lambda }{2}\mathop \sum \limits_{l = 1}^{{n_{l} - 1}} \mathop \sum \limits_{i = 1}^{{s_{l} }} \mathop \sum \limits_{j = 1}^{{s_{l} + 1}} \left( {W_{ji}^{l} } \right)^{2} \hfill \\ & = \left[ {\frac{1}{m}\mathop \sum \limits_{i = 1}^{m} \left( {\frac{1}{2}\left\| {h_{W,b} \left( {x^{\left( i \right)} } \right) - y^{\left( i \right)} } \right\|^{2} } \right)} \right] + \frac{\lambda }{2}\mathop \sum \limits_{l = 1}^{{n_{l} - 1}} \mathop \sum \limits_{i = 1}^{{s_{l} }} \mathop \sum \limits_{j = 1}^{{s_{l} + 1}} \left( {W_{ji}^{l} } \right)^{2} \hfill \\ \end{aligned}$$

where *m* is the number of training examples, $\lambda$ controls the relative importance of the second term, the first term of loss function (2) is an average sum-of-squares error term, the second term is the weight decay term which tends to decrease the magnitude of weights and prevent over-fitting.

### Continuous sparse autoencoder

In order to extract the features of nonlinear data, the zero-mean Gaussian with variance $\sigma^{2}$ stochastic unit is added into activation function of each visible unit.

3 $$s_{j} = \varphi_{j} \left( {\sum\limits_{i} {w_{ij} x_{i} + a_{i} + \sigma \cdot N_{j} \left( {0,1} \right)} } \right)$$

Equation (3) refers to the activation function with Gaussian stochastic unit, $\varphi_{j}$ represents the activation function, and $s_{j}$ is the output of network with input $x_{i}$, $a_{i}$ is the bias unit, $N_{j} \left( {0,1} \right)$ means a zero-mean Gaussian, $\sigma$ and $N_{j} \left( {0,1} \right)$ composes $n_{j} = \sigma \cdot N_{j} \left( {0,1} \right)$, $n_{j}$ subjects to the distribution as

4 $$p\left( {n_{j} } \right) = \frac{1}{{\sigma \sqrt {2\pi } }}\exp \left( {\frac{{ - n_{j}^{2} }}{{2\sigma^{2} }}} \right)$$

The unit activation of hidden layer can be defined as (Andrew 2012)

5 $$\hat{\rho }_{j} = \frac{1}{m}\mathop \sum \limits_{i = 1}^{m} \left[ {a_{j}^{\left( 2 \right)} \left( {x^{\left( i \right)} } \right)} \right]$$

where $a_{j}^{\left( 2 \right)} \left( {x^{\left( i \right)} } \right)$ means the activation of hidden layer unit with the input $x$, $\rho$ means the sparse parameter. In this paper, we assume that $\hat{\rho }_{j} { = }\rho$, the difference between $\hat{\rho }_{j}$ and $\rho$ can be calculated by Kullback–Leibler (KL) divergence (Kullback and Leibler 1951)

6 $${\text{KL}}(\rho | |\hat{\rho }_{j} )= \rho \log \frac{\rho }{{\hat{\rho }_{j} }} + \left( {1 - \rho } \right)\log \frac{1 - \rho }{{1 - \hat{\rho }_{j} }}$$

7 $$J_{sparse} \left( {W,b} \right) = J\left( {W,b} \right) + \beta \mathop \sum \limits_{{{\text{j}} = 1}}^{{s_{2} }} {\text{KL}}(\rho | |\hat{\rho }_{j} )$$

where $\beta$ is the weight coefficient that controls the sparse penalty factor. According to the loss function (1), suppose that $L_{2}$ is a hidden layer, $L_{1}$ represents the input layer and $L_{3}$ is the output layer, the error of output layer can be calculated

8 $$\delta_{\text{i}}^{\left( 3 \right)} = \frac{\partial }{{\partial z_{i}^{\left( 3 \right)} }}\frac{1}{2}\left\| {h_{W,b} \left( x \right) - y} \right\|^{2} = - \left( {y_{i} - a_{i}^{\left( 3 \right)} } \right)f^{\prime}\left( {z_{i}^{\left( 3 \right)} } \right)$$

where $\delta_{\text{i}}^{\left( 3 \right)}$ means the error of output layer, $a_{i}^{\left( 3 \right)}$ is the activation function, $z_{i}^{\left( 3 \right)} = W_{\text{i}}^{\left( 2 \right)} a_{i}^{\left( 2 \right)} + b^{\left( 2 \right)}$. In the hidden layer $L_{2}$, the error of each unit can be calculated as

9 $$\delta_{\text{i}}^{\left( 2 \right)} = \left( {\left( {\mathop \sum \limits_{j = 1}^{{s_{2} }} W_{ji}^{\left( 2 \right)} \delta_{\text{i}}^{\left( 3 \right)} } \right) + \beta \left( { - \frac{\rho }{{\hat{\rho }_{j} }} + \frac{1 - \rho }{{1 - \hat{\rho }_{j} }}} \right)} \right)f^{\prime}\left( {z_{i}^{\left( 2 \right)} } \right)$$

10 $$\frac{\partial }{{\partial W_{ji}^{\left( l \right)} }} J\left( {W,b;x,y} \right) = a_{j}^{\left( l \right)} \delta_{\text{i}}^{{\left( {l + 1} \right)}}$$

11 $$\frac{\partial }{{\partial b_{i}^{\left( l \right)} }} J\left( {W,b;x,y} \right) = \delta_{\text{i}}^{{\left( {l + 1} \right)}}$$

The gradient descent optimization parameters can be obtained:

1. Setting $$\varDelta W^{\left( l \right)} : = 0,\varDelta b^{\left( l \right)} : = 0$$
2. Calculating $$\nabla_{{W^{\left( l \right)} }} J\left( {W,b;x,y} \right)$$ and $$\nabla_{{b^{\left( l \right)} }} J\left( {W,b;x,y} \right)$$
3. Calculating $$\varDelta W^{\left( l \right)} : = \varDelta W^{\left( l \right)} + \nabla_{{W^{\left( l \right)} }} J\left( {W,b;x,y} \right)$$ and $$\varDelta b^{\left( l \right)} : = \varDelta b^{\left( l \right)} + \nabla_{{b^{\left( l \right)} }} J\left( {W,b;x,y} \right)$$
4. Updating the weight:
  $$W^{\left( l \right)} = W^{\left( l \right)} - \alpha \left[ {\left( {\frac{1}{m}\varDelta W^{\left( l \right)} } \right) + \lambda W^{\left( l \right)} } \right]$$
  $$b^{\left( l \right)} = b^{\left( l \right)} - \alpha \left[ {\left( {\frac{1}{m}\varDelta b^{\left( l \right)} } \right)} \right]$$

In this paper, manifold learning is drawn to analyze the effect of stochastic unit. According to the manifold learning theory, the high-dimensional data can be represented by low-dimensional manifold. The operator $p(x|\tilde{x})$ attempts to transform the high-dimensional $x$ to low-dimensional $\tilde{x}$. In the process of learning, the distribution of stochastic unit is not in high-dimensional manifold, so the gradient of $p(x|\tilde{x})$ should be changed greatly to approximate *x*. Essentially, CSAE can be considered as a manifold learning algorithm. The stochastic unit added into activation function can change the gradient direction and prevent over-fitting.

**Fig. 3:**
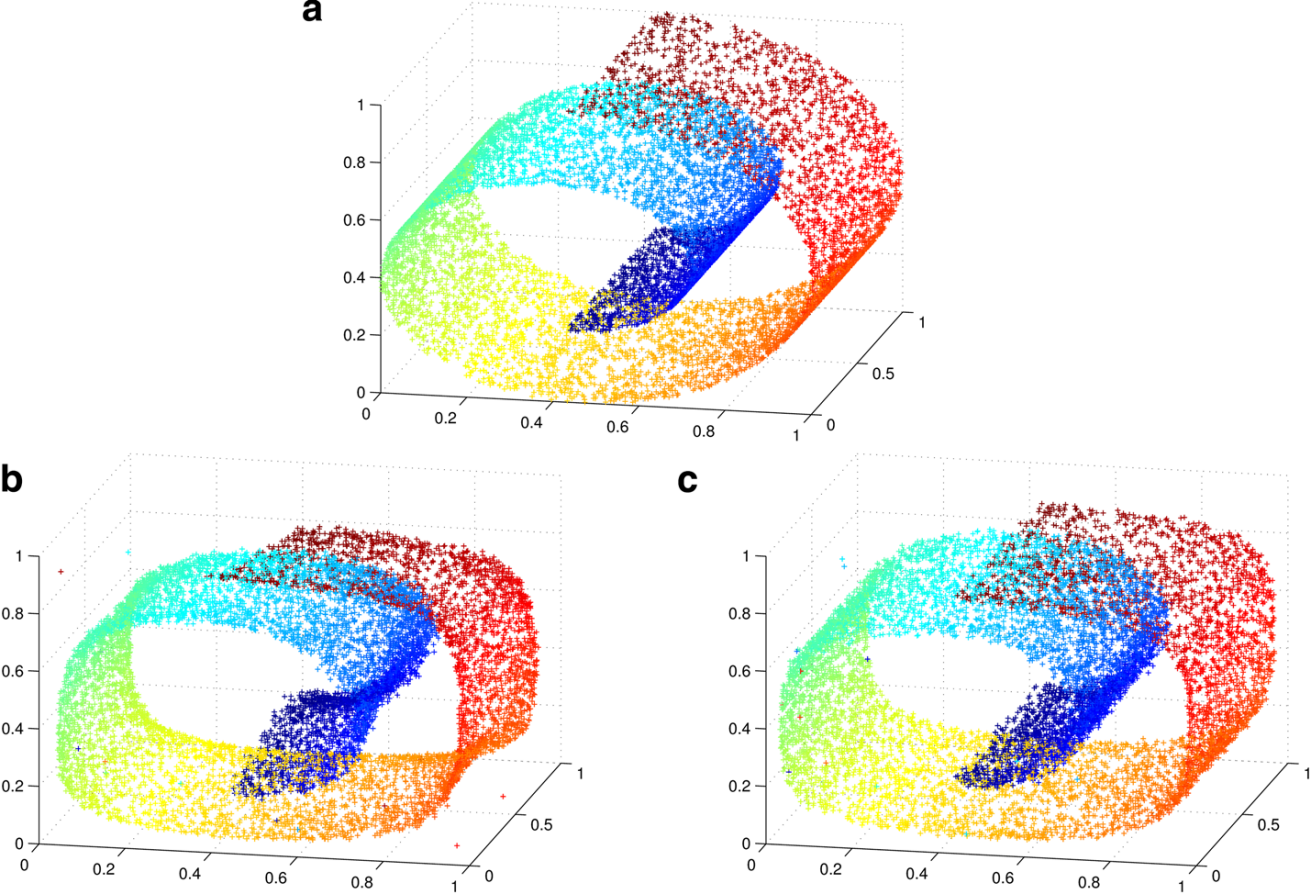
Reconstruction of swiss-roll mainfold **a** raw swiss-roll mainfold. **b** reconstruction of swiss-roll mainfold by autoencoder, **c** reconstruction of swiss-roll mainfold by CSAE

The contrast experiment of autoencoder and CSAE has been designed. The swiss-roll manifold is adopted as the experiment dataset. The result of experiment is shown in Fig. 3. Figure 3a is the raw swiss-roll manifold, Fig. 3b, c   are the reconstruction of swiss-roll dataset by autoencoder and CSAE. It can be concluded that CSAE is more suitable for reconstructed of continuous data than autoencoder.

## Experiments

### Dataset and normalization

In this paper we use IEC TC 10 as the experiment dataset (Duval and DePablo 2001) provided by Mirowski and LeCun (2012). There are 134 transformer fault samples in this dataset. Each sample contains the concentrations of CH_4_, C_2_H_2_, C_2_H_4_, C_2_H_6_ and H_2_ in parts per million (ppm). Three ratios including CH_4_/H_2_, C_2_H_2_/C_2_H_4_, C_2_H_4_/C_2_H_6_ can be calculated as the input of DBN. The five classifications of transformer faults corresponding to binary codes can be set as the output of DBN, they are 00001 (partial discharges), 00010 (low energy discharge), 00100 (high energy discharge), 01000 (thermal faults <700 °C) and 10000 (thermal faults >700 °C).

The concentrations of gases dissolved in transformer oil have a direct guiding significance for transformer fault analysis. In order to reduce the singularity of data and improve the training speed, the input data can be normalized to $\left[ {y_{min} ,y_{max} } \right]$ by normalization formula

12 $$y = \frac{{(y_{max} - y_{min} )\left( {x - x_{min} } \right)}}{{(x_{max} - x_{min} )}} + y_{min}$$

where $y$ represents the normalized data, making $y_{max} = 1$, $y_{min} = - 1$. $x_{max}$ is the maximum value of input data, while $x_{min}$ is the minimum value of input data.

### Network structure

**Fig. 4:**
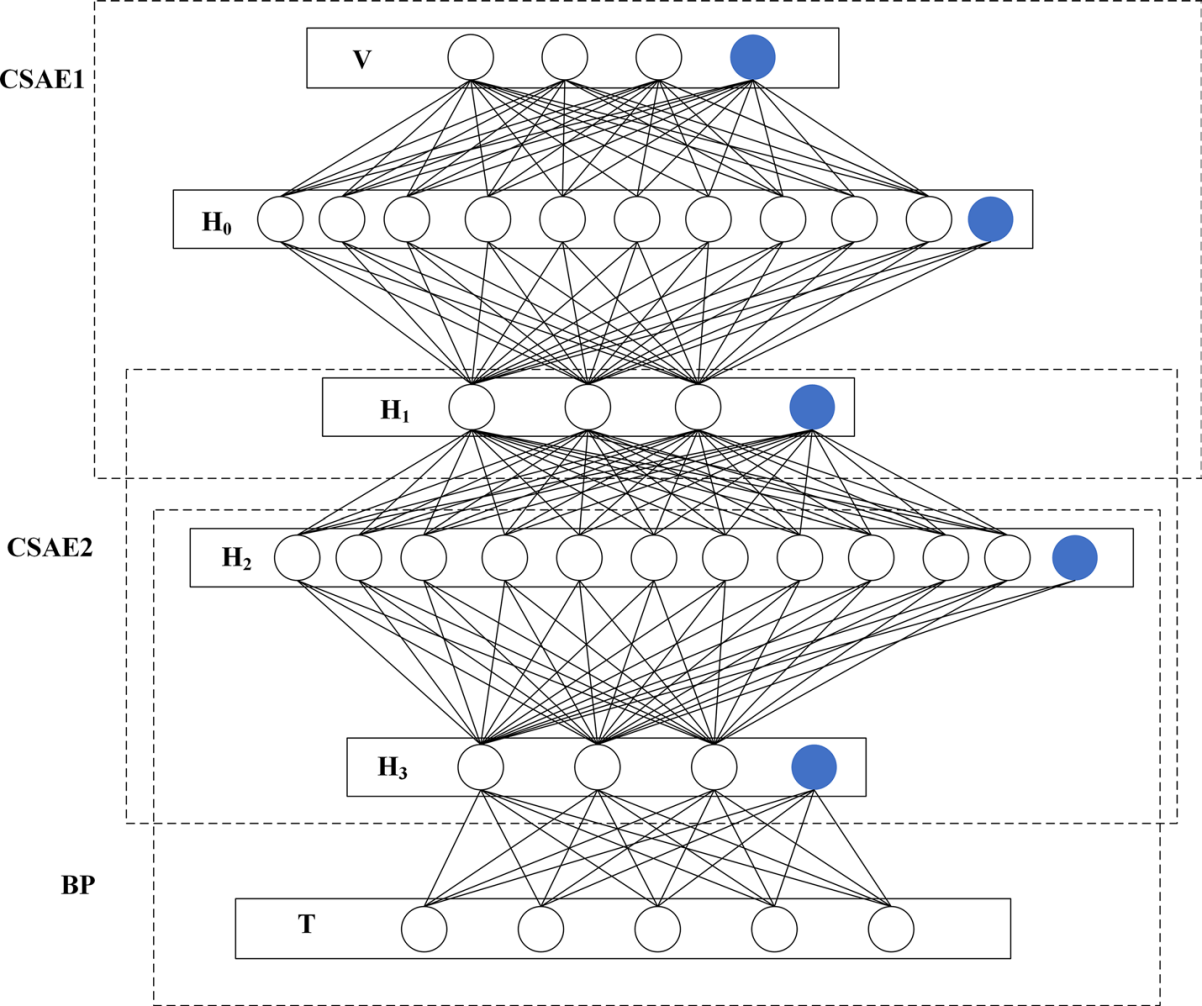
Network structure

The network structure is shown in Fig. 4. The white circles are neuron units and the blue circles denote bias units. There are six layers: the input layer V, the hidden layer H_0_, the hidden layer H_1_, the hidden layer H_2_, the hidden layer H_3_ and the output layer T. Layer V, layer H_0_ and layer H_1_ compose the first CSAE network. Layer H_1_, layer H_2_ and layer H_3_ compose the second CSAE network. Layer H_2_, layer H_3_ and layer T compose BP network. In layer V, there are 3 units (not including the bias unit, the same below) which contain three ratios of transformer fault gases. T layer contains 5 units corresponding to the transformer faults binary codes. The hidden layer H_0_ contains 10 units which are used to store the high-dimensional features. The hidden layer H_1_ contains 3 units which are used to reconstruct the high-dimensional features to low-dimensional approximate output. The hidden layer H_2_ and H_3_ contain 11 and 3 units respectively.

**Fig. 5:**
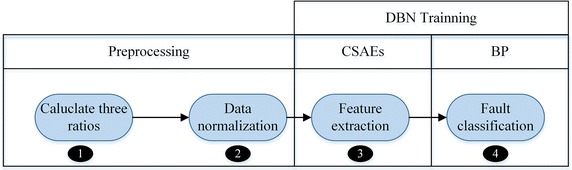
Flowchart of proposed method

The flowchart of proposed method is shown in Fig. 5. The phases of transformer fault diagnosis mainly include preprocessing and DBN training. In the preprocessing phase, the three ratios of transformer fault samples can be calculated. Then the data can be normalized by Eq. (12) as the input of DBN. In DBN training phase, two CSAEs are used to extract the hidden features of input, BP is used to reduce the dimension of hidden features and classify the transformer fault.

### Parameters setting

Parameters are very important for neural network. Recent studies (Nguyeny et al. 2013) have shown that if parameters is not set properly, the correct differentiation rate will be low and the speed of convergence will be slow. According to previous experience, the authors set parameters as follows.

Learning rate: the learning rate is very important. If it is big, the system will become unstable. Otherwise the training epoch will become too long. Generally, a relatively small learning rate will make the error converge asymptotically. At the same time, because the network size is different, the learning rate should be adjusted according to the network size. In this experiment, the learning rate is set to be 0.05.

Momentum: in order to avoid over-fitting and fine-tune the direction of gradient, we apply the momentum parameter to change the gradient of likelihood function. In this experiment, the momentum is set to be 0.9.

13 $$W_{ij} \leftarrow k \times W_{ij} + \epsilon \left({\left\langle {v_{i} h_{j}} \right\rangle_{data} - \left\langle {v_{i} h_{j}} \right\rangle_{reconstruct}} \right)$$

Sparse parameter: sparse parameter is used to determine the unit activation. In this experiment, the sparse parameter is set to be 0.01.

### Simulation

About the simulation environment, the software is Matlab 8.1.0 and the hardware is the desktop computer with Intel i5 processer with 8 GB RAM and 2.5 GHz frequency, and the operating system is Microsoft Windows 8.1 professional. In this experiment, the 125 samples are applied to the training dataset, and the other 9 samples are applied to the predicting dataset. The K-fold is adopted to the cross validation method. In this section, K is set to be 5, it means that 125 samples will be divided into 5 partitions. One partition is used for testing and the other 4 partitions are used for training. The process will repeat 5 times until each partition can be regarded as training and testing data.

**Fig. 6:**
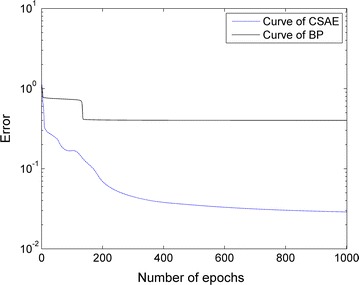
CSAE and BP error curve

Through training of 125 samples, Fig. 6 shows the error curve of CSAE and BP. It can be proved that the convergence speed of CSAE curve is faster than BP curve. And the error of CSAE curve is lower than BP curve.

In order to verify the validity of our approach, the classification accuracy of K-nearest neighbor (K-NN), support value machine (SVM), BP and CSAE are contrasted. Table 3 shows the classification accuracy of K-NN algorithm. When K = 15, the accuracy is 90 %. SVM is applied as one of the standard tool for pattern classification and recognition. SVM converts samples into a feature space using kernel functions which commonly include radial basis function (RBF), polynomial function (PLOY) and sigmoid function (SIG) (Hsu and Lin 2002). The classification accuracy of SVM with different kernel functions is shown in Table 4, the highest correct rate of SVM using RBF as the kernel function is 79.9 %.

**Table 3:** Classification accuracy of K-NN

K	10 (%)	15 (%)	20 (%)	60 (%)
Accuracy (%)	88.9	90	83.9	77.8

**Table 4:** Classification accuracy of SVM

Kernel function	SVM_RBF (%)	SVM_SIG (%)	SVM_PLOY (%)
Accuracy (%)	79.9	59.5	68.8

Through 1000 epochs training, Table 5 lists the correct differentiation rates of BP and CSAE. These two models have the same parameters of network which can ensure the fairness of results. The correct rate of BP algorithm is 86.6 % in TF1 and HED. The correct rate of CSAE algorithm is 100 % in TF1, 83.3 % in PD.

**Table 5:** Classification accuracy of BP and CSAE

Classification	CSAE (%)	BP (%)
TF1 (%)	100	86.6
TF2 (%)	93.7	81.2
PD (%)	83.3	83.3
LED (%)	95.6	82.6
HED (%)	95.5	86.6

Wilcoxon rank sum test (Wilcoxon 1945) is a well-known nonparametric statistical test used to evaluate the ranking of features. In this paper, the Wilcoxon rank sum test is used to compare the differences of CSAE and BP algorithm. It is assumed that h = 1 denotes the fact that the correct differentiation rate of CSAE is significantly better than BP; h = 0 denotes the fact that the correct differentiation rate of CSAE is as same as BP; the level of significance α = 0.05. The results are shown in Table 6. The average correct differentiation rate of CSAE and BP are 93.6 ± 6.22 and 84.1 ± 2.44 % respectively. The *p* value is 0.0195 which is smaller than α. So it can be concluded that the correct differentiation rate of CSAE is significantly better than BP.

**Table 6:** Results of Wilcoxon rank sum test

State	CSAE (%)	BP (%)
Standard deviation (%)	6.22	2.44
Average accuracy (%)	93.6	84.1
p-value	0.0195	

Based on the training network, 9 test samples are adopted to check the forecast ability of CSAE. In Table 7, it can be seen that the fault of CSAE algorithm forecast is consistent with the actual fault.

**Table 7:** A part of training results

No	CH_4_/H_2_	C_2_H_2_/C_2_H_4_	C_2_H_4_/C_2_H_6_	Actual fault	Forecast fault
1	0.06	0	1.35	LED	LED
2	1	0.007	2.52	TF1	TF1
3	0.96	0.025	8.12	TF2	TF2
4	2.3	0	3.83	TF2	TF2
5	7.19	0.005	8.63	TF2	TF2
6	0.235	1.1	7.67	PD	PD
7	1.3	0	1.22	TF1	TF1
8	1.23	0.05	9.22	TF2	TF2
9	0.17	1	9.615	PD	PD

## Conclusion and future work

In this paper, we propose a novel CSAE model which can be used in unsupervised learning of representations. CSAE added Gaussian stochastic unit in activation function is adopted to solve the problem of transformer fault recognition. The IEC three ratios are calculated by the concentrations of dissolved gases. Then the three ratios are normalized to reduce data singularity. In the experiments, DBN is established by two layers of CSAE and one layer of BP. CSAE is applied to unsupervised training and getting features. BP is used for supervised training and transformer fault classification. Comparative experiments clearly show the advantages of CSAE on transformer fault diagnosis. This neural network diagnosis algorithm is better than the traditional algorithm with its value in the actual transformer fault diagnosis.

The CSAE model have the advantages of outstanding recognition ability of continuous data, unsupervised feature learning ability, high precision and robust ability. The main disadvantages of CSAE model include long time training and high performance computer requirement. In summary, CSAE has great potential. In the future work, we will continue to research CSAE and try to use some tricks to shorten the training time. Furthermore, we plan to investigate some optimization strategies to diagnosis the transformer fault.
